# The coding potential of circRNAs

**DOI:** 10.18632/aging.101554

**Published:** 2018-09-13

**Authors:** Aniruddha Das, Myriam Gorospe, Amaresh C. Panda

**Affiliations:** 1 Institute of Life Sciences, Nalco Square, Bhubaneswar, Odisha, India; 2 Laboratory of Genetics and Genomics, National Institute on Aging, National Institutes of Health, Biomedical Research Center, Baltimore, MD 21224, USA

**Keywords:** circular RNA, ribosomes, translation, Cap-independent, m6A

Discovered three decades ago, circular (circ)RNAs were thought to be non-functional splicing artifacts, but it is now well established that they are generated by the covalent joining of 5’ and 3’ ends of exons and/or introns by a process known as backsplicing [1]. The biogenesis of circRNAs is modulated by different molecular factors, including RNA-binding proteins (RBPs), splicing components, proteins affecting transcription elongation, and the presence of inverted RNA repeats [1]. Until now, more than 100,000 circRNAs have been identified, many of them expressed in specific tissues and associated with distinct physiologic and pathologic states, including muscle aging [2]. The handful of circRNAs for which a function was identified have been shown to influence gene expression patterns by acting as sponges for microRNAs and RBPs [1]. Interestingly, while circRNAs are noncoding in nature, it was proposed early on that they could have coding potential, since many circRNAs originate form exons and reside in the cytoplasm, where they could be translated. However, only a handful of circRNAs have been reported to be translated into functional proteins.

Abe and colleagues were among the first to report the rolling circle translation of an artificial circRNA with infinite open reading frame (ORF) in rabbit reticulocyte lysate [3]. This study revealed that exonic circRNAs could be used by the eukaryotic translation machinery to produce proteins without the 3’ poly(A) or 5’ cap structures. Recently, several ribosome bound circRNAs (ribo-circRNAs) were identified in Drosophila by analyzing the circRNA reads with predicted ORFs across the backspliced junction sequences present in the ribosome footprint data [4]. To check the translatability of ribo-circRNAs, a minigene construct expressing circMbl was transfected into insect S2 cells and protein translation was assessed. Interestingly, the untranslated regions of the ribo-circRNAs (cUTRs) were found to be critical for the cap-independent translation of these circRNAs [4].

In another study, Yang et al. discovered that motifs for the most abundant RNA modification, N6-methyladenosine (m6A), were enriched in the circRNA population [5]. Interestingly, a single m6A modification was found to be enough to initiate circRNA translation requiring the initiation factor eIF4G2 and the m6A reader protein YTHDF3. Translation of circRNAs initiated by m6A was promoted by methyltransferase METTL3/14 and suppressed by demethylase FTO. The m6A-mediated translation of endogenous circRNAs was found to occur constitutively but was upregulated by heat shock [5]. Legnini et al. reported that endogenous circ-ZNF609 is expressed in murine and human myoblasts and translated to protein [6]. This translation is dependent on the splicing machinery but is cap-independent and can be modulated by stress. Although the product of circ-ZNF609 was not characterized functionally, it was found to promote myogenic proliferation.

These advances have begun to uncover new molecular strategies that can be used to express functional proteins for basic research, commercial applications, and clinical use. While earlier efforts to express functional protein from eukaryotic mRNAs were complicated by the fact that linear mRNAs are generally unstable, circRNAs containing functional ORFs can be used successfully to express proteins in far larger amounts. In this regard, a study by Wesselhoeft et al. reported a novel method to synthesize circRNAs *in vitro* using self-splicing introns with additional sequences that aid in splicing [7]. These synthetic circRNAs were found to be translated into large quantities of high-quality protein over extended times in eukaryotic cells [7].

Altogether, these findings strongly suggest that circRNAs can be exploited for the production of desired functional proteins. In the case of ectopic protein production from circRNAs for therapeutic purposes, delivery methods as well as precise timing and quantity of protein production need to be carefully developed and optimized. A deeper understanding of the features that improve production of proteins from circRNAs will enable their therapeutic use in many pathologies, including age-associated diseases in which the production of functional protein is impaired.
